# Facile Synthesis of Nitrogen-Doped Graphene Quantum Dots/MnCO_3_/ZnMn_2_O_4_ on Ni Foam Composites for High-Performance Supercapacitor Electrodes

**DOI:** 10.3390/ma17040884

**Published:** 2024-02-14

**Authors:** Di Liu, Soeun Kim, Won Mook Choi

**Affiliations:** 1 School of Chemical Engineering, University of Ulsan, 93 Daehak-ro, Nam-gu, Ulsan 44610, Republic of Korea; ldygyx1020@163.com (D.L.); soeundl12@naver.com (S.K.); 2 Division of Advanced Material, Korea Research Institution of Chemical Technology, 141 Gajeong-ro, Yuseong-gu, Daejeon 34114, Republic of Korea

**Keywords:** N-GQDs, MnCO_3_/ZnMn_2_O_4_, hydrothermal, hierarchical, supercapacitors

## Abstract

This study reports the facile synthesis of rationally designed composite materials consisting of nitrogen-doped graphene quantum dots (N-GQDs) and MnCO_3_/ZnMn_2_O_4_ (N/MC/ZM) on Ni foam using a simple hydrothermal method to produce high-performance supercapacitor applications. The N/MC/ZM composite was uniformly synthesized on a Ni foam surface with the hierarchical structure of microparticles and nanosheets, and the uniform deposition of N-GQDs on a MC/ZM surface was observed. The incorporation of N-GQDs with MC/ZM provides good conductivity, charge transfer, and electrolyte diffusion for a better electrochemical performance. The N/MC/ZM composite electrode delivered a high specific capacitance of 960.6 F·g^−1^ at 1 A·g^−1^, low internal resistance, and remarkable cycling stability over 10,000 charge–discharge cycles. Additionally, an all-flexible solid-state asymmetric supercapacitor (ASC) device was fabricated using the N/MC/ZM composite electrode. The fabricated ASC device produced a maximum energy density of 58.4 Wh·kg^−1^ at a power density of 800 W·kg^−1^ and showed a stable capacitive performance while being bent, with good mechanical stability. These results provide a promising and effective strategy for developing supercapacitor electrodes with a high areal capacitance and high energy density.

## 1. Introduction

At present, due to energy depletion and environmental degradation, researchers have begun to study new, efficient energy storage devices. Supercapacitors have attracted growing attention due to their rapid charging and discharging capability, impressive power density, safety features, and environmental sustainability [1,2,3,4]. Pseudocapacitors operate through the reversible redox reaction of the active materials, such as transition metal compounds, leading to better electrochemical properties than those of the electrochemical double-layer capacitors (EDLCs) that store the charges in an electric double layer [5,6,7]. The active material on the electrodes is the most significant part of the supercapacitor, and it directly determines the performance of the device. Therefore, it is very critical to prepare high-performance electrode materials for energy storage devices.

In recent years, a variety of pseudocapacitive electrode materials, such as transition metal oxides/hydroxides/sulfides/carbonate, have been used as the electrode material for high-performance capacitors to realize the practical application of supercapacitors [8,9,10,11,12]. Many binary transition metal oxides, such as ZnCo_2_O_4_, NiCo_2_O_4_, ZnMn_2_O_4_, and Sn_x-0_Mn_x_S, have been assessed as high-capacity electrodes for supercapacitors and Li-ion batteries because of their low cost, environmentally friendly nature, and very good electrochemical performance [13,14,15,16]. However, these transition metal oxide-based electrodes for supercapacitors show limited electrical conductivity and a specific capacity. To avoid these drawbacks, composites containing different oxides for supercapacitor electrode materials, such as ZnO@MnCo_2_O_4_, MnO_2_@CoNiO_2_, Fe_3_O_4_@Fe_2_O_3_, etc., have been designed and investigated to improve the electrochemical performance of supercapacitors [17,18,19]. Hu et al. reported the development of starfish-shaped porous Co_3_O_4_/ZnFe_2_O_4_ hollow nanocomposites on Ni foam using a simple hydrothermal method, which exhibited an excellent specific capacitance of 326 F·g^−1^ at 1 A·g^−1^ [20]. Another effective approach was the incorporation of carbon-based materials, including graphene, carbon nanotubes, and graphitic carbon nitride (g-C_3_N_4_), to enhance their conductivity. Hong et al. reported that unique Ni(OH)_2_ nanoflower/reduced graphene oxide composites were fabricated on carbon cloth for high-performance supercapacitor applications, and their composite electrode exhibited a high specific capacitance of 1900 F·g^−1^ at 1 A·g^−1^ [21]. Therefore, combining two types of binary metal compound and a carbon-based material is one of the best routes for improving the electrochemical performance.

In addition, graphene quantum dots (GQDs), as zero-dimensional graphitic nanocrystals with a size of only several nanometers, have been a focus in the electrochemical field, owing to their unique structure and superior properties. GQDs can be synthesized through a bottom-up approach using organic precursors and biomolecules, which are cost-effective and environmentally friendly. GQDs have much higher edge/core atomic ratios than the usual carbon materials do, which could greatly enhance the electrochemical activities of electrodes [22,23,24,25]. The electronic properties of GQDs can be simply controlled using the hetero atom doping method and functionalization. Doping N atoms on carbon materials has also been considered as an effective route to enhance the supercapacitive performance of carbon electrode materials by providing extra pseudocapacitance due to the faradaic interactions between electrolyte ions and the carbon surface. Therefore, N-doped GQDs (N-GQDs) can serve as highly electrochemically active carbon materials to combine with metal oxides for a strong capacitive performance [26,27,28,29].

In this study, novel hierarchical N-GQDs/MnCO_3_/ZnMn_2_O_4_ (N/MC/ZM) composites were directly synthesized on Ni foam using a simple hydrothermal method, followed by calcination to produce the high-capacitive electrodes of supercapacitors. The preparation of composite materials with different metal oxide compounds (MnCO_3_/ZnMn_2_O_4_) with carbon-based materials (N-GQDs) could result in enhanced capacitive performance and electrical conductivity. Moreover, the incorporation of N-GQDs provides an effective modification route for MC/ZM electrodes due to their simple and green synthesis. The N/MC/ZM composites exhibited excellent electrochemical properties, including a high specific capacitance of 960.6 F·g^−1^, with excellent cycling stability due to the incorporation of N-GQDs. In addition, a flexible all-solid-state asymmetric supercapacitor (ASC) was fabricated using the N/MC/ZM composite electrode. The prepared ASC device delivered a maximum energy density of 58.4 Wh·kg^−1^ at a power density of 800 W·kg^−1^, with good mechanical stability. The proposed N/MC/ZM composite provides an effective strategy for the development of high-capacitive and durable electrodes for supercapacitors.

## 2. Materials and Methods

### 2.1. Synthesis of N-GQDs

Typically, N-GQDs are synthesized by dissolving 0.21 g of citric acid (99%, Sigma-Aldrich, St. Louis, MO, USA) and 0.18 g of urea (99%, Samchun, Seoul, Republic of Korea) into 5 mL of deionized (DI) water and stirring to form a clear mixture solution. The resultant solution is then transferred into a 50 mL Teflon-lined stainless autoclave. The sealed autoclave is heated to 160 °C in an oven, and then a hydrothermal reaction occurs for 4 h. After the reaction, the autoclave reactor is cooled down to room temperature. The obtained aqueous mixture solution is centrifuged at 5000 rpm for 5 min to collect the upper layer. Furthermore, the obtained upper aqueous solution is dialyzed with a dialysis membrane (3500 Da, Spectrum Lab. Inc., Piscataway, NJ, USA) overnight, and the dialyzed aqueous solution is collected to obtain a pristine N-GQDs solution (2 mg·mL^−1^).

### 2.2. Preparation of MnCO_3_/ZnMn_2_O_4_ and N-GQDs/MnCO_3_/ZnMn_2_O_4_

**Figure 1:**
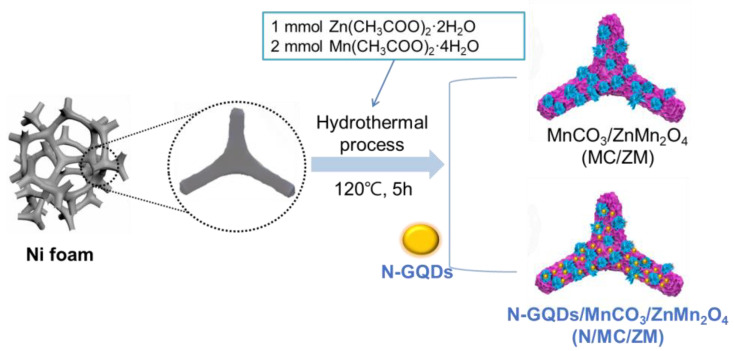
Schematic illustration for the synthesis of MC/ZM and N/MC/ZM.

The synthesis of the MnCO_3_/ZnMn_2_O_4_ (MC/ZM) and N-GQDs/MnCO_3_/ZnMn_2_O_4_ (N/MC/ZM) composites is schematically illustrated in Figure 1. Typically, 2 mmol of manganese (II) acetate tetrahydrate (Mn(CH_3_COO)_2_·4H_2_O, 99.99%, Sigma Aldrich, St. Louis, MO, USA) and 1 mmol of zinc (II) acetate dehydrate (Zn(CH_3_COO)_2_· 2H_2_O, ≥99.0%, Sigma–Aldrich. St. Louis, MO, USA) are dissolved in 70 mL of DI water, and then 6 mmol of ammonium fluoride (25%, Samchun, Seoul, Republic of Korea) is added into the solution. Then, 0.015 mol of urea is added into the solution mixture, which is stirred for 20 min. Then, this solution mixture and a piece of Ni foam (2 × 3 cm^2^) are transferred to a 90 mL hydrothermal autoclave reactor. The hydrothermal reaction is performed at 120 °C for 5 h in an oven. Then, the obtained Ni foam is cooled down to room temperature and repeatedly washed with DI water 5 times, and then dried at 60 °C in a vacuum oven. Finally, the Ni foam product is calcined at 350 °C for 2 h in the air to obtain the hierarchical MnCO_3_/ZnMn_2_O_4_ on Ni foam (MC/ZM). For the preparation of the N-GQDs/MnCO_3_/ZnMn_2_O_4_ composite (N/MC/ZM), this procedure was employed with adding 2 mL of N-GQDs (0.5 mg·mL^−1^) solution into the mixture solution for the hydrothermal reaction.

### 2.3. Characterizations

The chemical composition and crystal phase of MC/ZM and N/MC/ZM on Ni foam were characterized with X-ray powder diffraction using a Bruker D8 Advance diffractometer with non-monochromated Cu Kα operated at 40 kV and 30 mA. The X-ray photoelectron spectroscopy (XPS: Thermo Fisher, Waltham, MA, USA) measurements were performed using monochromatic AlKα radiation (hν = 1486.6 eV). The morphology of MC/ZM and N/MC/ZM on Ni foam was tested using field emission scanning electron microscopy (JEOL FESEM-JSM820), and energy dispersive X-ray spectroscopy (EDS) was also performed with the attached SEM instrument. High-resolution transmission electron microscopy (HRTEM) and selected area electron diffraction (SAED) observations were carried out (JEOL JEM-F200) at 200 kV.

### 2.4. Electrochemical Measurements

The electrochemical performance was investigated using cyclic voltammetry (CV), galvanostatic charge/discharge (GCD), and electrochemical impedance spectroscopy (EIS) tests. All the electrochemical measurements were performed using a BioLogic VSP electrochemical workstation at room temperature. A three-electrode system was used to test the electrochemical performance of the prepared electrodes with platinum foil and Hg/HgO electrodes as the counter and reference electrodes, respectively. The Ni foam covered with MC/ZM and N/MC/ZM was cut into small pieces, and the loading density of MC/ZM and N/MC/ZM on the Ni foam was calculated to be around 1.5–1.7 mg·cm^−2^; a small piece was used directly as a working electrode. All the electrochemical tests were performed in an aqueous electrolyte of 1 M KOH at room temperature. EIS was performed at an open circuit potential in the frequency range from 10 kHz to 0.01 Hz with an AC amplitude of 5 mV.

For the fabrication of an asymmetric supercapacitor (ASC), the solid-state flexible ASC was prepared using the MC/ZM and N/MC/ZM as the positive and negative electrodes, respectively, which were assembled with a PVA/KOH gel electrolyte and filter paper as a separator. The prepared two electrodes were wetted with the PVA/KOH gel electrolyte for 10 min, and then assembled face-to-face with filter paper using gentle pressure. The specific capacitance ($C_{s}$) of the product was calculated using the CV curves and galvanostatic charge–discharge curves according to the following equation:

(1) $$C_{s}=\frac{\int I\left(V\right)\;dV}{\nu \times m\times ∆V}$$

(2) $$C_{s}=\frac{I\times ∆t}{m\times ∆V}$$

where $\int I\left(V\right)\;dV$ represents the area enclosed by the CV curves, ΔV (V) represents the potential window, *ν* (mV·s^−1^) represents the potential scan rate, m (g) represents the mass of the active materials in the electrodes, and Δt (s) represents the discharge time. The energy density E (Wh·kg^−1^) and power density P (W·kg^−1^) of the device were calculated using the following equations:

(3) $$E=\frac{Cs\times {\left(∆V\right)}^{2}}{2\times 3.6}$$

(4) $$P=\frac{3600\times E}{∆t}$$

## 3. Results and Discussion

**Figure 2:**
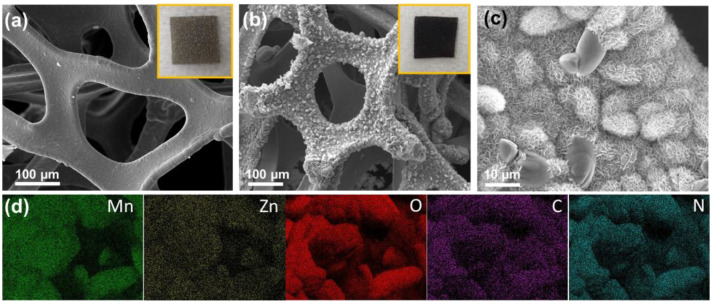
FESEM images of (**a**) pristine Ni foam, (**b**) N/MC/ZM on Ni foam (insets are photographs), and (**c**) highly magnified image of N/MC/ZM on Ni foam. (**d**) EDS elemental mapping of N/MC/ZM composite.

The synthesis of the MC/ZM and N/MC/ZM composites is schematically illustrated in Figure 1. The microstructures of the synthesized N/MC/ZM composites were characterized using FESEM. Figure 2a shows the typical morphology of the three-dimensional conductive framework structure of Ni foam, which can support the electrodes, allowing the transmission of electrons and ions, and can enhance the conductivity of the materials. After the synthesis of the N/MC/ZM composite, the hierarchical microstructure of the N/MC/ZM composite grew uniformly on the surface of Ni foam using a simple one-step hydrothermal method. This is also confirmed by the black colored surface of the N/MC/ZM composites on the gray colored pristine Ni foam, as shown in the inset photographs. Figure 2c represents the microstructure of MC/ZM synthesized on Ni foam. It is clear that the hierarchical microparticles with nanosheets grew uniformly on the substrate surface of the Ni foam. The nanometer-thick sheets grew on the whole frame of the Ni foam. These microstructures of N/MC/ZM composites can facilitate the diffusion of ions, charge transfer, and the infiltration of electrolytes, indicating that the electrochemical performance could be improved. In addition, the interconnected nanosheets provide a large surface area, which accommodates the strain induced by the volume change in the charge–discharge process. Due to the low content of N-GQDs, the deposition of N-GQDs was confirmed by FESEM measurement. For a further investigation of the existence of N-GQDs in the composite, EDS elementary mapping was conducted. As shown in Figure 2d, the uniform distribution of Mn, O, Zn, C, and N elements can be observed in the N/MC/ZM composites. Specifically, the uniform existence of C and N atoms clearly indicates the successful deposition of N-GQDs in the composite. However, the FESEM and EDS elementary mapping of MC/ZM show a similar microstructure, with the existence of only Mn, O, and Zn atoms (Figure S1 in the Supplementary Materials). In addition, the specific surface areas of N/MC/ZM were measured using N_2_ adsorption–desorption isotherms (Figure S2 in the Supplementary Materials). The calculated surface area was determined to be 276.4 m^2^ g^–1^. These results demonstrate the successful preparation of hierarchical N/MC/ZM composites through a facile one-pot hydrothermal process.

The phase structure of synthesized N/MC/ZM composites was investigated using XRD analysis. As shown in Figure S3 (in the Supplementary Materials), two distinct phases of diffraction peaks can be observed in the N/MC/ZM composites, which could be assigned to the phases of ZnMn_2_O_4_ (JCPDS NO.71-2499) and MnCO_3_ (JCPDS NO.83-1763). In detail, the typical peaks at 2θ = 18.2°, 29.3°, 33.0°, 36.0°, 44.7°, 51.9°, 54.4°, 56.7°, 59.0°, 60.8°, 61.9°, 65.1°, 68.3°, 75.0°, and 78.6° were assigned to the ZnMn_2_O_4_ phase, and the peaks at 2θ = 24.3°, 31.5°, 41.6°, and 45.4° correspond to the MnCO_3_ phase. These results also imply the formation of a mixed phase of MnCO_3_ and ZnMn_2_O_4_ in the N/MC/ZM composites. Also, the characteristic peaks of N-GQDs were not found due to their low content in the composites.

**Figure 3:**
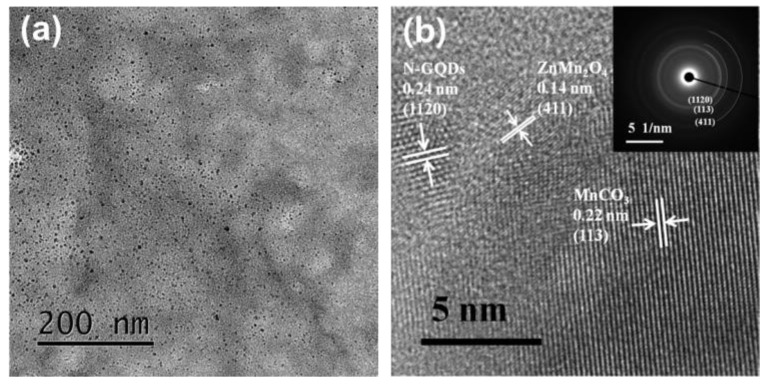
(**a**) TEM image and (**b**) HRTEM images of N/MC/ZM composite (insert shows the corresponding SAED patterns).

The morphology of the N/MC/ZM composites was further studied using TEM measurement. The TEM image of N/MC/ZM composites in Figure 3a shows that the N-GQDs are decorated on the nanosheets of MC/ZM. The detail, the HRTEM image in Figure 3b, also clearly exhibits the crystalline lattices of N-GQDs, ZnMn_2_O_4_, and MnCO_3_, which represent a graphene (1120) plane with a lattice distance of 0.24 nm, a MnCO_3_ (113) plane with a lattice distance of 0.22 nm, and a ZnMn_2_O_4_ (411) plane with a lattice distance of 0.14 nm. The SAED pattern of N/MC/ZM composites in the inset shows the polycrystalline ring corresponding to the (1120), (113), and (411) crystal planes of the composite. These results strongly indicate the successful decoration of N-GQDs into the MC/ZM structures.

**Figure 4:**
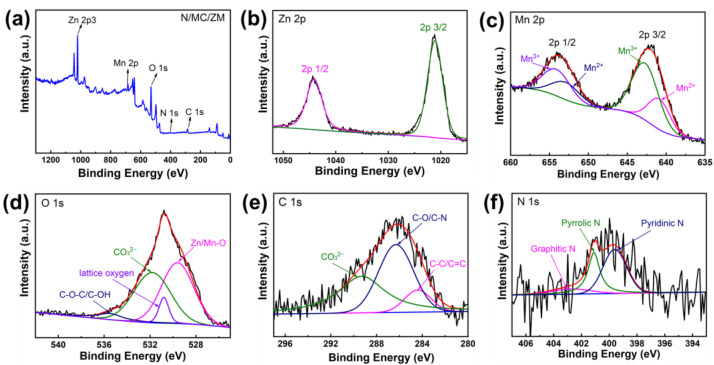
(**a**) XPS survey spectra, (**b**) Zn 2p, (**c**) Mn 2p, (**d**) O 1s, (**e**) C 1s, and (**f**) N 1s of N/MC/ZM composite.

XPS measurements were performed for further analysis of the surface chemical composition of the N/MC/ZM composite, and the obtained XPS spectra are displayed in Figure 4. The full survey spectrum of N/MC/ZM in Figure 4a exhibits the Zn, Mn, O, N, and C elements detected, suggesting that the N-GQDs were successfully doped on MC/ZM. To study the chemical states of the N/MC/ZM composite in detail, the high-resolution XPS spectra of Zn 2p, Mn 2p, O 1s, C 1s, and N 1s were investigated. As illustrated in Figure 4b, the high-resolution spectra of Zn 2p show two distinct peaks centered at 1021.2 and 1044.3 eV, corresponding to Zn 2p3/2 and Zn 2p1/2, respectively, with an energy difference of 23.1 eV, which is in good agreement with the Zn^2+^ oxidation states [30,31]. The Mn 2p high-resolution spectra presented in Figure 4c show two major peaks located at 642.1 and 653.7 eV, assigned to Mn 2p3/2 and Mn 2p1/2, respectively. These fitting peaks at 640.9 and 652.9 eV correspond to the Mn^2+^ oxidation state. The two peaks at 643 eV and 654.1 eV are related to the Mn^3+^ oxidation state [32]. In Figure 4d, the O 1s spectra display four fitting peaks at 529.6, 531.3, 531.8, and 535.5 eV, corresponding to the representative metal–oxygen bond, lattice oxygen, CO_3_^2−^, and C-O-C/C-OH, respectively [33,34,35,36]. These results indicate the successful synthesis of MnCO_3_/ZnMn_2_O_4_. Figure 4e shows the high-resolution spectra of C 1s of the N/MC/ZM composite. Three major peaks at 284.7, 286.3, and 289.3 eV can be clearly observed, which are attributed to the aromatic C-C/C=C, C-O/C-N, and CO_3_^2−^ functional groups, respectively. These results indicate the successful doping of N-GQDs on the N/MC/ZM composite [37]. The N 1s spectra of the N/MC/ZM composite in Figure 4f could be deconvoluted into three peaks centered at the binding energies of 399.6, 401.1, and 402.7 eV, ascribed to pyridinic N, pyrrolic N, and graphitic N, respectively. In these three nitrogen bonding configurations, pyrrolic N is in an sp^3^-hybridized state, and pyridinic N is in an sp^2^-hybridized state, which are both negatively charged. However, graphitic N is in a positively charged and sp^2^-hybridized state. Therefore, pyrrolic N and pyridinic N can generate pseudocapacitance via Faraday reactions, while graphitic N can enhance the conductivity of graphene materials through its positive charge and repair effect on graphene sheets. It is noteworthy that the results from the deconvoluted high-resolution spectra of O 1s, C 1s, and N 1 s indicate the presence of hydrophilic functional groups in the N/MC/ZM composite, which could feature pseudocapacitive behavior, particularly in the presence of aqueous electrolytes, to enhance the electrochemical performance of the N/MC/ZM composite [38,39].

**Figure 5:**
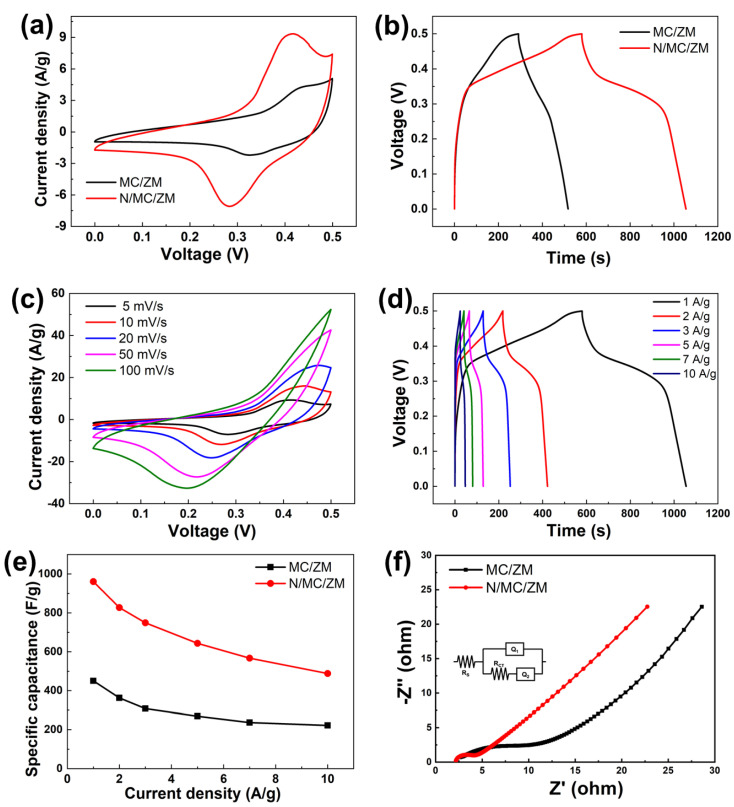
(**a**) CV curves of MC/ZM and N/MC/ZM electrodes at the scan rate of 5 mV·s^−1^ in 1.0 M KOH electrolyte. (**b**) GCD curves of MC/ZM and N/MC/ZM electrodes at 1 A·g^−1^ current density. (**c**) CV curves of N/MC/ZM electrode at different scan rates of 5–100 mV·s^−1^. (**d**) GCD curves of N/MC/ZM electrode at different current densities of 1–10 A·g^−1^. (**e**) The specific capacitance of MC/ZM and N/MC/ZM electrodes at different current densities derived from GCD. (**f**) Nyquist plots of MC/ZM and N/MC/ZM electrodes in 1.0 M KOH electrolyte and its equivalent circuit model.

The electrochemical performances of N/MC/ZM electrodes were investigated using Cyclic voltammogram (CV), galvanostatic charge–discharge (GCD), and electrochemical impedance measurements. Figure 5a shows the CV curves of the capacitive performance of the MC/ZM and N/MC/ZM electrodes in the potential window ranging from 0 to 0.5 V at a scan rate of 5 mV·s^−1^. The current and storage capacity were significantly enhanced for the N/MC/ZM electrode due to the addition of N-GQDs, compared to those of the MC/ZM electrode. The incorporation of N-GQDs facilitates proton or alkaline ion diffusion into the electrode, leading to enhanced Faradic reactions. The improved supercapacitor properties in the N/MC/ZM electrode mode are distinctly evident in the different voltammograms of the MC/ZM electrode. Both the MC/ZM and N/MC/ZM electrodes show well-defined redox peaks in the CV curves, where the redox peaks can be observed at the potentials of 0.42 and 0.31 V for the MC/ZM electrode, whereas the peaks can be observed at 0.41 and 0.29 V for the N/MC/ZM electrode. It is evident that EDLC materials will display rectangular curves, while transition metal oxides will manifest reversible redox peaks, indicating the Faradaic oxidation and reduction processes occurring on the material’s surface. Also, the pseudocapacitor materials show the characteristics of both the EDLC and metal oxides. In the prepared electrodes, the peaks at 0.42 and 0.41 V are attributed to oxidation, and the peaks at 0.31 and 0.29 V are ascribed to reduction. Within the pseudocapacitor materials, electrochemical reactions may take place through surface de/adsorption (or de/intercalation), alongside Faradaic redox reactions [40,41]. The electrochemical reaction of the N/MC/ZM electrode can be written as:

(5) $${\mathrm{ZnMn}}_{2}O_{4}+{\mathrm{OH}}^{-}+H_{2}O↔\mathrm{ZnOOH}+2\mathrm{MnOOH}+e^{-}$$

(6) $$\mathrm{MnOOH}+{\mathrm{OH}}^{-}↔{\mathrm{MnO}}_{2}+H_{2}O+e^{-}$$

(7) $${MnCO}_{3}+2{OH}^{-}\to Mn(OH{)}_{2}+{{CO}_{3}}^{2-}$$

(8) $$Mn(OH{)}_{2}+{OH}^{-}↔MnOOH+H_{2}O+e^{-}$$

(9) $$MnOOH+{OH}^{-}↔{MnO}_{2}+H_{2}O+e^{-}$$

(10) $${ZnMn}_{2}O_{4}+K^{+}+e^{-}↔\left[{ZnMn}_{2}O_{4}\right]K$$

(11) $${MnCO}_{3}+K^{+}+e^{-}↔\left[{MnCO}_{3}\right]K$$

Figure 5b represents the GCD curves of the MC/ZM and N/MC/ZM electrodes measured at a current density of 1 A·g^−1^ within the potential window of 0–0.5 V. With the N/MC/ZM composite electrode, a longer discharge time and larger specific capacitance value were observed than those of the MC/ZM electrode, according to Equation (2). The enhanced capacitive performance of the MC/ZM electrode can be ascribed to the presence of N-GQDs combined with MC/ZM, which promotes ion diffusion and transport capabilities at the electrode/electrolyte interface. This synergy leads to improved conductivity and a more efficient charge–discharge process. CV curves of N/MC/ZM with different scan rates ranging from 5 to 100 mV·s^−1^ in the potential range of 0–0.5 V are shown in Figure 5c. It can be observed that the current density exhibits a simultaneous increase with a higher scan rate, and the cyclic voltammetry (CV) curves characterized by symmetric redox peaks indicate high reversibility in the charge–discharge reaction. Furthermore, as the scan rate increases, the potentials of both the anodic and cathodic peaks shift towards more positive and negative values, respectively. This phenomenon can be attributed to the polarization effect and ion diffusion. A similar CV behavior was also observed with the same CV measurements for the MC/ZM electrode (Figure S4a in the Supplementary Materials); however, the enclosed area of the CV curves for the MC/ZM electrode are smaller than that of the N/MC/ZM electrode. The GCD curves of the prepared electrodes at different current densities from 1 to 10 A·g^−1^ were measured under a voltage ranging from 0 to 0.5 V. As shown in Figure 5d, the GCD curves obtained at the different current densities exhibit a well-defined potential plateau, which diminishes at a high current density of 10 A·g^−1^. This observation suggests the pseudocapacitive nature of the MC/ZM electrode. The GCD curves of the N/MC/ZM electrode retain a similar shape, with a small IR drop, implying good electrical conductivity and excellent capability. The specific capacitance values of the MC/ZM and N/MC/ZM electrodes were calculated from the GCD curves using Equation (2). As shown in Figure 5e, higher calculated specific capacitances are obtained for the N/MC/ZM composite electrodes, with 960.6, 827.5, 748.7, 643.4, 566.9, and 488.1 F·g^−1^ at current densities of 1, 2, 3, 5, 7, and 10 A·g^−1^, respectively, compared to those of the MC/ZM electrode (Figure S4b in the Supplementary Materials). The observed specific capacitance of the N/MC/ZM composite was compared to that of ZnMn_2_O_4_ based on Table 1, which reveals the superior specific capacitance of the prepared N/MC/ZM composite [31,42,43,44,45,46]. Also, the prepared N/MC/ZM composite presents a superior capacitive performance in comparison with those of the other reported Mn- and Zn-based electrodes (Table S1 in the Supplementary Materials) [47,48,49,50,51,52]. Additionally, the specific capacitances of five different N/MC/ZM composites were measured at 1 A·g^−1^ to investigate their reproducibility. Figure S5 (in the Supplementary Materials) exhibits the stable specific capacitance results for different N/MC/ZM electrodes, suggesting the easy reproducibility of the N/MC/ZM composite. Furthermore, Dunn and Trasatti’s method was employed to study the charge storage of the N/MC/ZM electrode as a pseudocapacitive energy storage material, in which the charge is stored via battery-like redox reactions at the interface between the electrolyte and active material. Due to the charge storage process, including adsorption/desorption and the intercalation/deintercalation of ions at the surface of active materials, the capacitance contribution is composed of EDLC (capacitive) and Faradic behaviors, corresponding to the outer ($C_{o}$) and inner charge storages ($C_{i}$), respectively. The charge captured on the surface $C_{o}$ of the electrode was calculated using the following equation:

(12) $$C\left(V\right)=a\nu^{-1/2}+C_{o}$$

The total stored charge ($C_{T}$) value was determined using the following equation:

(13) $$\frac{1}{C}=b\nu^{-1/2}+\frac{1}{C_{T}}\;$$

(14) $$C_{T}=C_{o}+C_{i}$$

where ν represents the scan rate (V·s^−1^), and C (C·g^−1^) = S/(ν × m) represents specific capacitance (S represents the integral area of CV curve at scan rate ν, and m represents the mass of active materials loaded on the electrode). By using Figure S6 (in the Supplementary Materials), the $C_{T}$ and $C_{o}$ of the N/MC/ZM electrode were calculated to be 481.2 and 385.04 C·g^−1^, respectively. C_o_ contributes around 80% of the total capacitance and 20% of the diffusive contribution, indicating a high active surface area for pseudocapacitance. The excellent storage performance is mainly due to the synergistic effect of the advantageous hierarchical structure of N-GQDs and MC/ZM, which includes enhanced mass transport, meso–macro-porosity, and well-aligned hierarchical nanostructures. To further explore the electrochemical kinetics, electrochemical impedance spectroscopy (EIS) was performed in the frequency range of 0.01 Hz–10 kHz at 5 mV amplitude and with an open circuit potential. Figure 5f shows Nyquist plots of the MC/ZM and N/MC/ZM electrodes with an equivalent circuit, where R_s_ and R_ct_ represent the ionic resistance of the electrolyte solution and its charge-transfer resistance at the contact interface between the electrode and electrolyte, respectively. The Nyquist plots of the MC/ZM and N/MC/ZM electrodes present a semicircle in the high-frequency region and a linear line in the low-frequency region. In the Nyquist plots, an intersection at the real axis at a high frequency describes the R_s_ of the electrochemical system, and the diameter of the semicircle represents the R_ct_ value. The straight-line slope indicates the diffusive resistance, which is related to the penetration of electrolyte ions. The N/MC/ZM electrode exhibits a smaller semicircle radius in the high-frequency region and a more vertical line in the low-frequency region compared to those of the MC/ZM electrode, demonstrating a lower Rct value (1.12 Ω) and the more rapid diffusion of electrolyte ions than those of the MC/ZM electrode (4.02 Ω). The fitted parameter from the EIS measurement is summarized in Table S2 (in the Supplementary Materials). Also, the obtained Nyquist plot was compared using the Kramers–Kronig transform method (Figure S7 in the Supplementary Materials), where similar plots suggest stable, accurate, and time-invariant EIS measurements in this study. This is ascribed to the incorporation of N-GQDs, facilitating improved ion transfer throughout the electrode and compensating for the low conductivity of MC/ZM.

**Figure 6:**
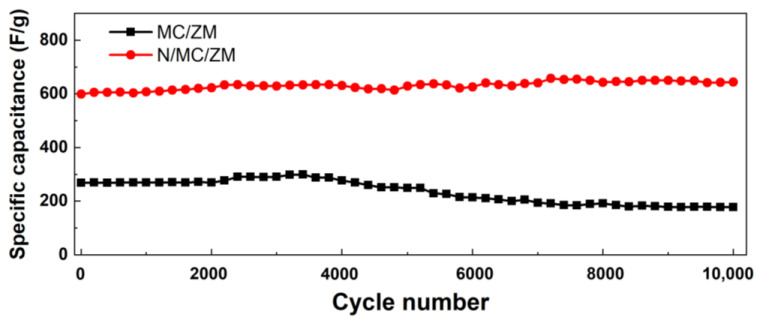
Cycling performance of MC/ZM and N/MC/ZM composites at 5 A·g^−1^.

The cycle stability of the N/MC/ZM composite electrode was further studied for 10,000 consecutive cycles at a current density of 5 A·g^−1^ (Figure 6). The N/MC/ZM electrode demonstrates remarkable preservation of capacitance over 10,000 cycles, while the capacitance of the MC/ZM electrode decreased after 4000 cycles. These results show the excellent cycle stability of the N/MC/ZM composite electrode.

**Figure 7:**
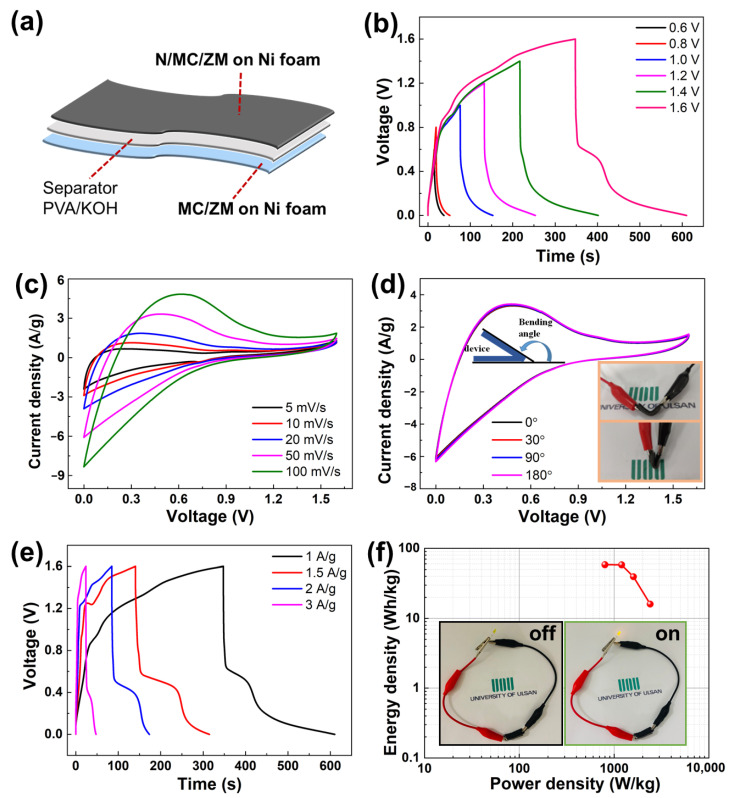
Two-electrode electrochemical performance of solid-state asymmetric supercapacitor (ASC) using N/MC/ZM//MC/ZM electrodes with PVA/KOH electrolyte. (**a**) Schematic illustration of flexible solid-state ASC device using N/MC/ZM//MC/ZM electrodes. (**b**) GCD curves of the ASC device at different potential windows at a current density of 1 A·g^−1^. (**c**) CV curves at different scan rates, and (**d**) CV curves under different bending conditions at 50 mV·s^−1^. (**e**) GCD curves of the fabricated ASC device at different current densities. (**f**) Ragone plots of the prepared ASC device using N/MC/ZM//MC/ZM electrodes. (Inset shows photographs of the LED light powered by the ASC device.)

In order to further investigate the practical application of the prepared MC/ZM and N/MC/ZM on Ni foam, a flexible solid-state asymmetric supercapacitor (ASC) was fabricated. In ASC device configuration, the MC/ZM and N/MC/ZM electrodes on Ni foams were assembled as the cathode and anode, with a piece of filter paper and polyvinyl alcohol (PVA)/KOH gel as a separator and electrolyte, respectively, as illustrated in Figure 7a. As depicted in Figure 7b, the GCD curves of the ASC device were first collected at 1 A g^−1^, with an operational voltage window ranging from 0.6 to 1.6 V, demonstrating that the voltage of the as-assembled ASC device using MC/ZM//N/MC/ZM electrodes can be stabilized up to 1.6 V. As shown in Figure 7c, the CV curves of the ASC using MC/ZM//N/MC/ZM exhibit capacitive characteristics at varied scan rates ranging from 5 to 100 mV·s^−1^. Specifically, a bending test was performed to assess the mechanical stability of the prepared ASC device. The CV curves in Figure 7d exhibit minimal changes, and capacitance retentions persist at approximately 100% across various bending angles from 0 to 180 degrees. This suggests the electrochemical durability and mechanical integrity of the ASC of the MC/ZM and N/MC/ZM electrodes. In addition, Figure 7e exhibits the GCD curves of the ASC devices at different current densities from 1 to 3 A·g^−1^. The calculated specific capacitance of the ASC using MC/ZM and N/MC/ZM electrodes was determined to be 164.3 F·g^−1^ at 1 A·g^−1^. Figure 7f further presents the power and energy densities of the ASC device of the MC/ZM//N/MC/ZM electrodes in a Ragone plot, where the prepared ASC device delivers a high energy density of 58.4 Wh·kg^−1^ at a power density of 800 W·kg^−1^. To demonstrate the need for a specific operating voltage and energy output for practical applications, the ASC device of the MC/ZM//N/MC/ZM electrodes turned on a commercial yellow LED (inset of Figure 7e). These results imply the ultrahigh energy density, flexibility, and practical application of the ASC of the MC/ZM//N/MC/ZM electrodes for high-performance energy storage.

## 4. Conclusions

The hierarchical structure of a N/MC/ZM composite electrode was prepared using a simple and versatile hydrothermal method for supercapacitor applications. N-GQDs were uniformly embedded in a hierarchical structure of MC/ZM, where the microparticles with nanosheets were synthesized on the Ni foam surface. The incorporation of N-GQDs in the N/MC/ZM composite shows synergistic effects, including more conductivity and rapid electron and ion transportation for a superior electrochemical performance. Therefore, the prepared N/MC/ZM composite electrode exhibited an enhanced specific capacitance of 960.6 F·g^−1^ at 1 A·g^−1^, with a good capability and excellent stability for 10,000 cycles. Additionally, the assembled ASC device using the N/MC/ZM electrode showed a maximum energy density of 58.4 Wh·kg^−1^ at a power density of 800 W·kg^−1^, with excellent mechanical stability. This study not only offers perspectives on high-capacitive supercapacitor electrodes, but also holds promise for enhancing the practical performance of supercapacitor devices.

## Figures and Tables

**Table 1:** The specific capacitance comparison of N/MC/ZM composite with the other reported ZnMn_2_O_4_-based electrodes.

**Material**	**Electrolyte**	**Specific Capacitance**	**Ref.**
ZnMn_2_O_4_ Nanofibers	1 M Na_2_SO_4_	240 F·g^−1^ @ 1 A·g^−1^	[42]
ZnMn_2_O_4_ Nanosheets	0.5 M Na_2_SO_4_	456.8 F·g^−1^ @ 1 A·g^−1^	[43]
ZnMn_2_O_4_/Mn_3_O_4_ composite	2 M KOH	380 F·g^−1^ @ 0.5 A·g^−1^	[31]
ZnMn_2_O_4_/Mn_3_O_4_ composite	1 M Na_2_SO_4_	321 F·g^−1^ @ 1 mV·s^−1^	[44]
ZnMn_2_O_4_ microspheres	1 M KOH	447 F·g^−1^ @ 1 A·g^−1^	[45]
Porous ZnMn_2_O_4_	6 M KOH	411.7 F·g^−1^ @ 1 A·g^−1^	[46]
N-GQDs/MnCO_3_/ZnMn_2_O_4_	1 M KOH	960.6 F·g^−1^ @ 1 A·g^−1^	This study

## Data Availability

The data presented in this study are available upon request from the corresponding author.

## Supplementary Materials

The following supporting information can be downloaded at: https://www.mdpi.com/article/10.3390/ma17040884/s1, Figure S1: FESEM image and its EDS elemental mapping images of MC/ZM on Ni foam; Figure S2: Nitrogen adsorption–desorption isotherms of N/MC/ZM; Figure S3: (XRD patterns of N/MC/ZM composite; Figure S4: (a) CV curves of MC/ZM at different scan rates and (b) GCD curves of MC/ZM at dif-ferent current densities; Figure S5: The calculated specific capacitances of different N/MC/ZM electrode samples; Figure S6: Linear fit of (a) C^−1 ^as function of v^1/2^ and (b) C as function of v^−1/2^; Figure S7: Nyquist plots measured using PEIS technique and Kramers-Kronig transforms; Table S1: The specific capacitance comparison of N/MC/ZM composite with other Mn^−^ and Zn based-electrodes; Table S2: Fitted EIS values for the components of the Nyquist equivalent circuit. References [47,48,49,50,51,52] were mentioned in the Supplementary Materials.
