# Supplementary material for: Facile Synthesis of Nitrogen-Doped Graphene Quantum Dots/MnCO3/ZnMn2O4 on Ni Foam Composites for High-Performance Supercapacitor Electrodes
Source: Materials (Basel). 2024 Feb 14;17(4):884. doi: 10.3390/ma17040884 (PMC10890349; doi:10.3390/ma17040884)
Supplement: Supplementary file 1 [file materials-17-00884-s001.zip › materials-2845395-supplementary.pdf]

materials

Supplementary Materials

Facile Synthesis of Nitrogen-Doped Graphene Quantum Dots/MnCO3/ZnMn2O4 on Ni Foam Composites for High-Performance Supercapacitor Electrodes

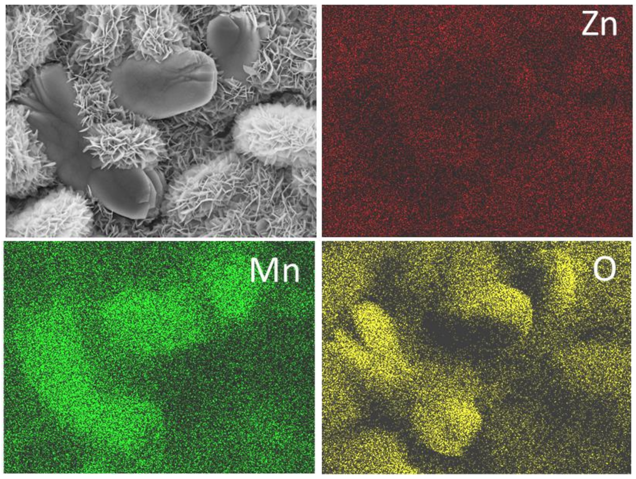

Figure S1. FESEM image and its EDS elemental mapping images of MC/ZM on Ni foam.

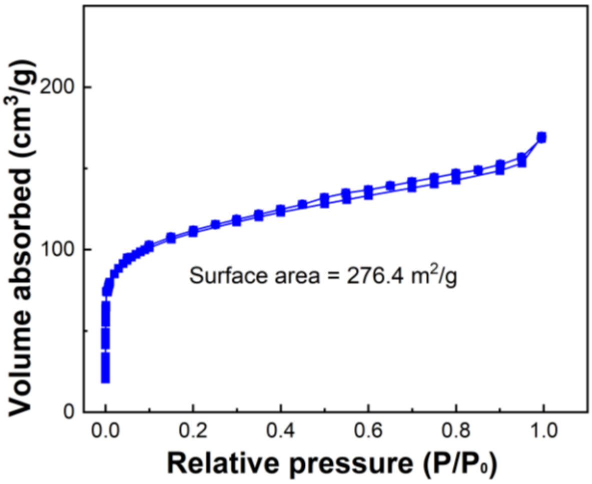

Figure S2. Nitrogen adsorption–desorption isotherms of N/MC/ZM.

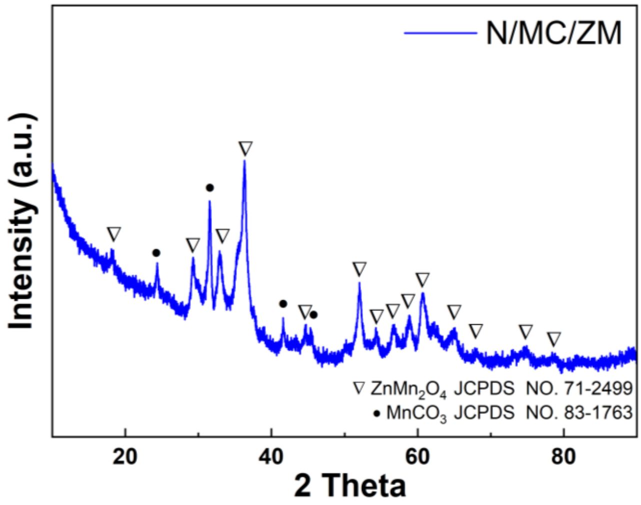

Figure S3. XRD patterns of N/MC/ZM composite.

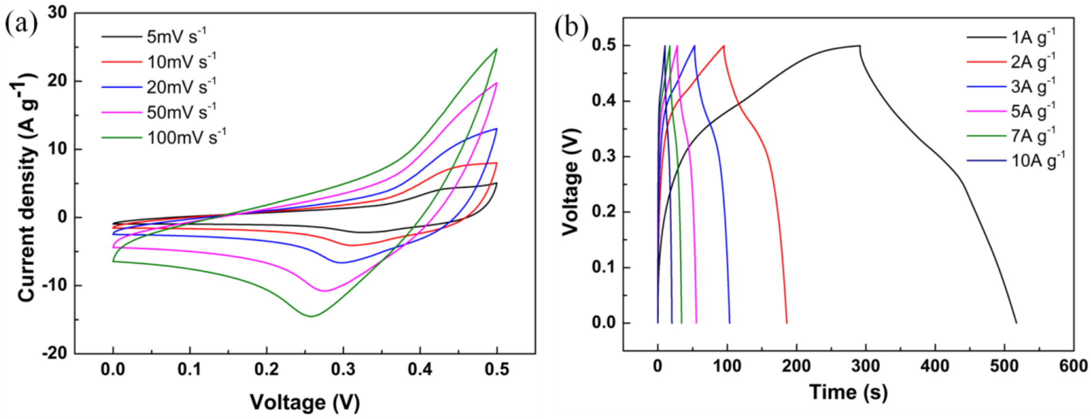

Figure S4. (**a**) CV curves of MC/ZM at different scan rates and (**b**) GCD curves of MC/ZM at different current densities.

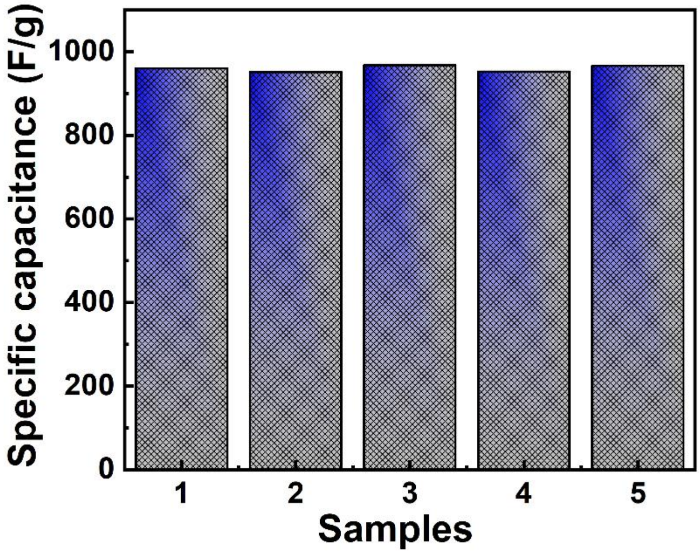

Figure S5. The calculated specific capacitances of different N/MC/ZM electrode samples.

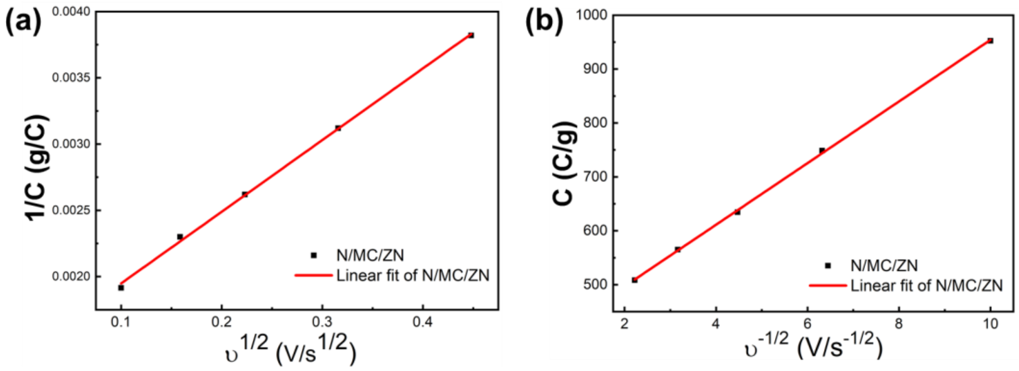

Figure S6. Linear fit of (**a**) C^{-1} as function of $v^{1/2}$ and (**b**) C as function of $v^{-1/2}$.

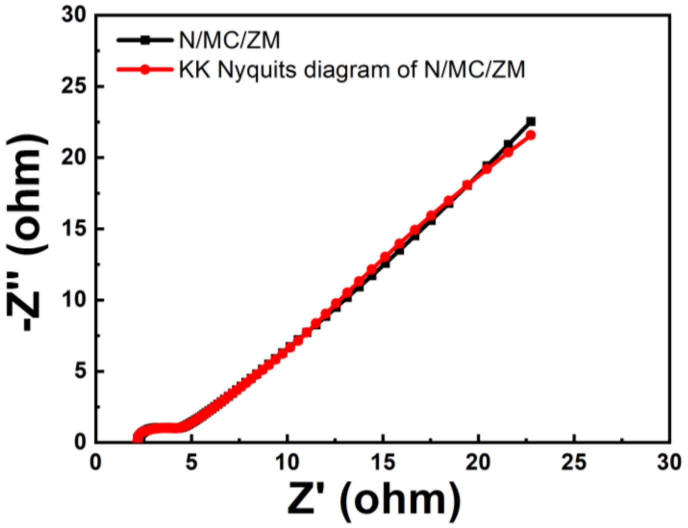

Figure S7. Nyquist plots measured using PEIS technique and Kramers-Kronig transforms.

Table S1. The specific capacitance comparison of N/MC/ZM composite with other Mn- and Zn based-electrodes.

Materials	Electrolyte	Specific Capacitance	Ref
Porous hollow double-walled Mn_2O_3 nanocubes	1 M KOH	256.25 F·g^{-1} @ 1 A g^{-1}	[47]
Carbon- MnO_x	6 M KOH	213.7 F·g^{-1} @ 0.5 A g^{-1}	[48]
K-dope MnO_2	6 M KOH	726 F·g^{-1} @ 1 A g^{-1}	[49]
ZnO/activated carbon	1 M Na_2SO_4	298 F·g^{-1} @ 1 A g^{-1}	[50]
ZnO nanorod/MnO_2 nanowires	1 M KOH	304 F·g^{-1} @ 1 A g^{-1}	[51]
polyindole/NiO/ZnO	1.0 M H_2SO_4	310.9 F·g^{-1} @ 0.5 A g^{-1}	[52]
N-GQDs/$MnCO_3$/$ZnMn_2O_4$	1 M KOH	960.6 F·g^{-1} @ 1 A g^{-1}	This study

Table S2. Fitted EIS values for the components of the Nyquist equivalent circuit.

Component	N/MC/ZM	MC/ZM
R_s (Ω)	2.14	2.48
R_{ct} (Ω)	1.12	4.02
Q_1 (F·s^{-1})	0.009	0.002
Q_2 (F·s^{-1})	0.749	0.401
